# Compressive Strength of Glass Ionomers With Different Polymerization Mechanisms: A Comparative In Vitro Analysis

**DOI:** 10.7759/cureus.93090

**Published:** 2025-09-24

**Authors:** Andrea C Merino, Jorge I Fajardo, Cesar A Paltan, Carlos Albán, Danny Espana

**Affiliations:** 1 Pediatric Dentistry, Universidad Hemisferios, Quito, ECU; 2 New Materials and Transformation Processes Research Group (GiMaT), Universidad Politécnica Salesiana, Cuenca, ECU; 3 Dentistry, Universidad Nacional de Chimborazo, Riobamba, ECU; 4 Oral Rehabilitation, Universidad Hemisferios, Quito, ECU

**Keywords:** compressive strength, dental materials, glass ionomer cements, light-curing, pediatric dentistry, self-curing

## Abstract

Aim: The aim of the study is to evaluate the compressive strength of different glass ionomer cements with distinct polymerization mechanisms through an in vitro experimental design.

Methods: Four materials were analyzed: GC Fuji IX GP Fast GIC (GC Corporation, Tokyo, Japan) and Maxxion R FGM (FGM, Joinville, Brazil) (self-curing) and GC Fuji II LC (GC Corporation) and Riva Light Cure SDI (SDI, Bayswater, Australia) (light-curing). One hundred cylindrical specimens (25 per material) were prepared following ISO 9917-1:2007 standards and stored in artificial saliva at 37°C for 15 days before undergoing compression tests.

Results: Statistically significant differences (p < 0.001) were found between materials, with Riva Light Cure SDI exhibiting the highest compressive strength (1,603.7 kN) and tensile strength (121.47 MPa), which were significantly superior to those of other materials. Surprisingly, no significant differences (p = 0.911) were found between GC Fuji IX GP Fast (self-curing, 911.2 kN) and GC Fuji II LC (light-curing, 965.8 kN), suggesting that the manufacturer's specific chemical composition had a greater influence than the polymerization mechanism. Maxxion R FGM presented the lowest values (613.0 kN), but with less variability.

Conclusion: Glass ionomer cements can be classified into three performance categories: high (Riva Light Cure SDI), intermediate (GC products), and basic (Maxxion R FGM), providing a framework for clinical selection based on specific requirements. The statistical equivalence between GC products with different polymerization mechanisms offers valuable flexibility for diverse clinical situations in pediatric dentistry.

## Introduction

Glass ionomer cements (GICs) were developed by Chaudhary et al. [1] in 1969 in response to the need to combine the advantages of silicate cements and polycarboxylate cements. The original GICs were based on an acid-base reaction between polyacrylic acid and fundamental glass particles, specifically alumina-fluoro-silicate glass [2]. This initial composition allowed for materials with chemical adhesion to the dental structure and fluoride release, highly desirable characteristics in restorative dentistry [3].

The capacity of GICs for chemical adhesion to dental structure, fluoride release, and biocompatibility positioned GICs as versatile materials for various clinical applications [4]. Over the last few decades, these materials have undergone significant modifications in their composition and polymerization mechanisms, seeking to optimize both their physical properties and clinical applications [3].

Since their introduction in the 1970s, GICs have undergone numerous modifications to their composition, aimed at enhancing their clinical characteristics [2]. Initially, they presented significant limitations, including a paste that thickened rapidly during manipulation, prolonged setting times, and unsatisfactory mechanical resistance. These drawbacks motivated research oriented toward optimizing both the composition and polymerization mechanisms [5].

The chemical composition of GIC powder has been significantly modified in recent times to improve its handling characteristics and mechanical properties [1]. Chaudhary et al. proposed that greater poly-salt bridges could be formed in the GIC matrix by increasing the chemical affinity between filler particles and the matrix, thus overcoming many existing obstacles; this chemical evolution was fundamental for expanding the spectrum of clinical applications of these materials [6].

A significant qualitative leap occurred with the introduction of nanotechnology in 2007, which was applied to both conventional ionomer cements and resin-modified versions [6]. The incorporation of nanoparticles, known as nanomers and nanoclusters, in fluoro-aluminosilicate (FAS) glass substantially improved the physical and mechanical properties of conventional glass ionomers [1]. This technological advancement represented one of the most promising developments in enhancing the resistance characteristics of these materials [6].

The development of resin-modified glass ionomers marked another significant milestone in their evolution [2]. These materials, while maintaining the elemental composition of conventional ionomers, incorporated HEMA (2-hydroxyethyl methacrylate) monomers and the camphorquinone initiator as fundamental components [6]. This modification enabled the combination of traditional acid-base reactions with photocuring polymerization, significantly improving mechanical properties and expanding the range of clinical applications [2].

Over time, GICs have enormously diversified their clinical applications, becoming versatile materials used in a wide variety of dental procedures [3]. Currently, they are employed as restorative materials, cementation agents, cavity bases and liners, pit and fissure sealants, endodontic sealers, and materials for atraumatic restorative treatment [7]. They are considered materials of choice for many specific procedures, including the sandwich technique, treatment of root caries, stress-free reconstructions, and long-term provisional restorations [3].

The most recent conceptual evolution led to considering glass ionomers as "smart materials," defined as those whose properties can be altered in a controlled manner by various stimuli such as stress, temperature, humidity, pH, or electric and magnetic fields [8]. This characterization reflects the capacity of these materials to interact with the oral environment, particularly through fluoride release and recharge, providing therapeutic effects that transcend the simple restorative function [9].

GICs can be broadly classified into conventional, resin-modified, and nano-enhanced types, each with distinct characteristics and applications [10]. Traditional glass ionomers are composed of basic aluminosilicate glass and polyacrylic acid, which harden through an acid-base reaction. They are used in dental fillings, adhesive cements, and atraumatic restorative procedures [1]. However, they exhibit poor mechanical properties and are sensitive to moisture, which has prompted various modifications [3].

Resin-modified glass ionomers incorporate resin components to improve aesthetics and reduce setting time. They offer better mechanical properties and moisture resistance compared to conventional GICs, making them suitable for areas requiring greater strength and aesthetic appeal [6]. Nano-enhanced glass ionomers incorporate nanoparticles, such as nano-hydroxyapatite and silica, to enhance stability and biocompatibility [10]. Among their advantages are the improvement of mechanical properties and the enhancement of dentinogenic differentiation potential. Ongoing studies focus on strengthening the bioactivity and antibacterial properties of materials through nanotechnology [11].

The classification of GICs based on their polymerization mechanisms enables an understanding of their biomechanical behavior and clinical applications [2]. Conventional GICs, also called self-curing or auto-curing, represent the first generation of these materials [12]. Their elemental composition consists of a powder of FAS glass particles and a liquid composed mainly of polyacrylic acid [13]. The setting mechanism is based exclusively on an acid-base reaction that occurs when both components are mixed, without requiring any external stimulus. This reaction produces the formation of a polycarboxylate matrix cross-linked with metal ions from the glass [14]. Conventional GICs have had a prolonged presence in restorative dentistry, being traditionally used as materials for cavity liners, cavity bases, direct restorations, and cementation agents [13].

Resin-modified GICs (RMGICs) emerged as a response to the limitations of conventional ionomers, particularly their sensitivity to moisture during the initial setting stage and their limited mechanical properties [4]. These materials maintain the essential components of traditional GICs (basic glass powder, water, and polyacid) but also incorporate a monomer component, typically HEMA, and a photosensitive initiator system, such as camphorquinone [12]. The setting mechanism of RMGICs is dual, combining the traditional acid-base reaction (neutralization) with addition polymerization activated by visible light [13]. This modification significantly overcame handling difficulties, reduced initial hardening time, and substantially improved wear resistance and general mechanical properties of the cement [4].

The most recent evolution in this classification is represented by triple-cure GICs, which represent an additional refinement of RMGICs [12]. These materials are based on more complex formulations that include copolymers of acrylic-itaconic acid with methacrylate groups bonded to the amide, in addition to HEMA, tartaric acid, and water [15]. Their setting mechanism is particularly sophisticated, initiating with an acid-base reaction when mixing the components, followed by a double polymerization reaction: one initiated by light (photopolymerization) and another chemically activated (self-polymerization) [12]. This triple polymerization pathway aims to optimize the material's properties and mitigate its limitations, leveraging the advantages of each mechanism [15].

The clinical relevance of compressive strength is crucial in evaluating and selecting ionomers, particularly for restorative applications. This physical property enables the prediction of the clinical behavior and longevity of dental restorations, particularly when using materials with varying polymerization mechanisms. The ability of a restorative material to withstand forces generated during mastication is fundamental to its clinical performance during masticatory function; dental restorations are subjected to compressive loads that can exceed 800 MPa in certain areas [16]. High compressive strength enables the restoration to effectively resist pressure and repetitive stress generated during mastication, phonation, and other functional oral activities, thereby preventing fractures and deformations that could compromise the restoration's integrity [17].

Compressive strength is considered a reliable indicator of a restoration's durability, establishing a direct correlation between this property and clinical survival time [18]. Longitudinal studies have demonstrated that the increase in compressive strength of glass ionomers significantly enhances their long-term resistance, thereby improving their performance as restorative materials, particularly in situations of high functional demands, such as restorations in posterior teeth [19].

Compressive strength tests are of special importance because they more faithfully simulate the stress pattern experienced during functional masticatory activities [2]. Unlike other mechanical properties, compressive strength constitutes the only indicator that can precisely identify the actual capacity of a restoration to withstand occlusal forces, providing clinically relevant information about its potential in vivo performance [7].

Various factors significantly influence the compressive strength of glass ionomers, including their chemical composition, the powder/liquid ratio employed, and the method of material manipulation [20]. The different polymerization mechanisms modify the final structure of the matrix, directly affecting its mechanical properties [21]. Additionally, clinical procedures such as applying a protective coating after the restoration can increase compressive strength, thereby optimizing the biomechanical behavior of the material [16].

One of the historical limitations of conventional glass ionomers has been their low resistance to crack propagation when exposed to high occlusal forces, restricting their use as direct restorative materials in extensive cavities subjected to load. This characteristic has motivated a constant search for formulations with improved mechanical properties, with the optimization of compressive strength being a fundamental requirement for expanding their clinical application in areas of functional load support [22].

In specific procedures such as atraumatic restorative technique (ART), the use of a glass ionomer with optimal mechanical properties is determinant for therapeutic success [23]. High-viscosity materials, which allow a certain degree of packability, have demonstrated favorable clinical results in multiple longitudinal trials [5]. The compressive strength of these materials directly correlates with their clinical survival rate, validating the importance of this property as a predictor of long-term functional behavior [24]. Therefore, this study aimed to evaluate the compressive strength of different GICs with distinct polymerization mechanisms through an in vitro experimental design.

## Materials and methods

This in vitro comparative experimental study evaluated two self-cured GICs (GC Fuji IX GP Fast GIC (GC Corporation, Tokyo, Japan) and Maxxion R FGM (FGM, Joinville, Brazil)) and two light-cured cements (GC Fuji II LC (GC Corporation) and Riva Light Cure SDI (SDI, Bayswater, Australia)). Dimensional verification and energy calibration were performed using precision instruments to ensure methodological consistency.

Specimen preparation followed the parameters defined by ISO 9917-1:2007 [11]. The sample size was calculated using G*Power 3.1.1 (Heinrich-Heine-Universität Düsseldorf, Düsseldorf, Germany), with a power of 95% and a significance level of 5%, resulting in a minimum of 100 samples. The effect size calculation was based on preliminary data from pilot studies and previous literature on comparisons of GIC compressive strength. Using Cohen's f for one-way ANOVA, we anticipated a large effect size (f = 0.4) based on expected mean differences of approximately 400 kN between material groups with pooled standard deviations (SDs) of 300 kN. The analysis parameters were as follows: α = 0.05, power (1-β) = 0.95, number of groups = 4, and effect size f = 0.4. The calculated total sample size was 92 specimens, rounded up to 100 (25 per group) to account for potential specimen losses during preparation and testing. This effect size was pre-specified based on clinical relevance thresholds where differences greater than 300 kN in compressive strength would be considered clinically meaningful for restorative material selection. Standardized cylindrical specimens (4 mm diameter × 6 mm height) were obtained using stainless steel molds. Celluloid strips and a glass slab ensured smooth, flat surfaces without irregularities.

Material manipulation respected the manufacturers’ instructions. For light-cured ionomers, a VALO curing unit (Ultradent Products, Inc., South Jordan, UT, USA) was used, with radiant output confirmed using a digital radiometer (exceeding 1,400 mW/cm²), which was monitored before each cycle [3].

Samples were stratified into two main groups according to polymerization mechanism: Group Ga (n = 50): self-cured ionomers: Ga1: GC Fuji IX GP Fast GIC (n = 25) and Ga2: Maxxion R FGM (n = 25); Group Gb (n = 50): light-cured ionomers: Gb1: GC Fuji II LC (n = 25) and Gb2: Riva Light Cure SDI (n = 25). Only specimens meeting the inclusion criteria, with dimensional accuracy (4 mm × 6 mm) and no visible surface defects, were included in the analysis. Defective specimens were excluded [11].

Conditioning was performed following ISO 9917-1:2007 [11]. Specimens were immersed in artificial saliva and stored in a precision incubator at 37°C ± 1°C for 15 days, protected from light. Initial and final pH values of each unit were recorded [12]. Storage was conducted at the Clinical Laboratory of the National University of Chimborazo, with all variables (temperature, medium composition, and duration) systematically documented. Mechanical property testing was conducted at GiMaT (New Materials and Transformation Processes Research Group, Salesian Polytechnic University), under controlled environmental conditions (23°C ± 1°C, 50% ± 5% RH), following international standards.

Compressive strength was tested as described by Panetta et al. [9] using a Shimadzu AGS-X Universal Testing Machine (Shimadzu Corporation, Kyoto, Japan) (20 kN load cell, 1.0 mm/min speed). Load was applied along the sample’s longitudinal axis, with flat ends placed between the compression plates. Maximum fracture force and other mechanical parameters (elastic modulus, energy to fracture) were recorded using Trapezium X software (Shimadzu Corporation) [9], ensuring real-time acquisition and analysis.

Statistical analysis was performed using SPSS v27.0 (IBM Corp., Armonk, NY, US). One-way ANOVA with post hoc comparisons was used to assess differences between groups, followed by non-parametric independent t-tests where appropriate [11].

Statistical significance was established at an α level of 0.05 for all analyses. Before parametric testing, data normality was assessed using the Shapiro-Wilk test, and homogeneity of variances was evaluated using Levene's test. Due to violations of normality assumptions, the non-parametric Kruskal-Wallis test was employed for group comparisons, followed by pairwise comparisons using Dunn's test with Bonferroni correction for multiple comparisons. Effect sizes were calculated using eta-squared (η²) for Kruskal-Wallis tests, with η² ≥ 0.01, ≥0.06, and ≥0.14 representing small, medium, and significant effects, respectively.

## Results

Table 1 shows the descriptive values of the maximum force measurements achieved by type of cement, finding marked differences in compressive strength. Riva Light Cure SDI significantly stood out with an average maximum force of 1,603.7 kN, representing approximately 66% of the sample.

**Table 1:** Descriptive statistics of maximum force

Material	Mean force (kN)	Standard deviation	Minimum force (kN)	Maximum force (kN)
GC Fuji IX GP Fast GIC	911.169	287.551	623.61	1,198.72
Maxxion R FGM	612.984	162.423	450.56	775.407
GC Fuji II LC	965.819	197.052	768.76	1,162.87
Riva Light Cure SDI	1,603.7	417.158	1,186.54	2,020.85

The variability observed through SD showed that Riva Light Cure SDI, despite its higher resistance, also presented the greatest dispersion of results (SD = 417.158 kN). In contrast, Maxxion R FGM, although exhibiting the lowest resistance values (mean = 612.984 kN), presented the least variability (SD = 162.423 kN).

The similarity in performance between GC Fuji II LC (965.819 kN) and GC Fuji IX GP Fast (911.169 kN) was notable, with a difference of only 5.7% in their means, despite employing different polymerization mechanisms. A notable finding was the similarity in mechanical behavior between GC Fuji II LC and GC Fuji IX GP Fast, with maximum force values of 965.8 and 911.2 kN (Table 1), respectively, and maximum tensions of 73.2 and 69.0 MPa (Table 2). Maxxion R FGM consistently showed the lowest values in both maximum force (613.0 kN) (Table 1) and maximum tension (46.4 MPa) (Table 2).

**Table 2:** Descriptive statistics of maximum tension

Material	Mean tension (MPa)	Standard deviation	Minimum tension (MPa)	Maximum tension (MPa)
GC Fuji IX GP Fast GIC	69.01	21.30	25.27	108.16
Maxxion R FGM	46.43	12.03	27.72	78.84
GC Fuji II LC	73.15	14.62	52.32	114.23
Riva Light Cure SDI	121.47	30.96	67.81	174.34

Table 2 shows a distribution pattern similar to that observed in maximum force. Riva Light Cure SDI presented the highest tensile resistance (121.47 MPa), approximately 66% higher than the average of the other three materials.

The amplitude of the range between minimum and maximum values varied considerably among the materials. GC Fuji IX GP Fast showed the highest relative range, with a maximum value (108.16 MPa) that quadrupled its minimum value (25.27 MPa). GC Fuji II LC presented the narrowest relative range, with a maximum value (114.23 MPa) approximately double its minimum value (52.32 MPa).

**Figure 1:**
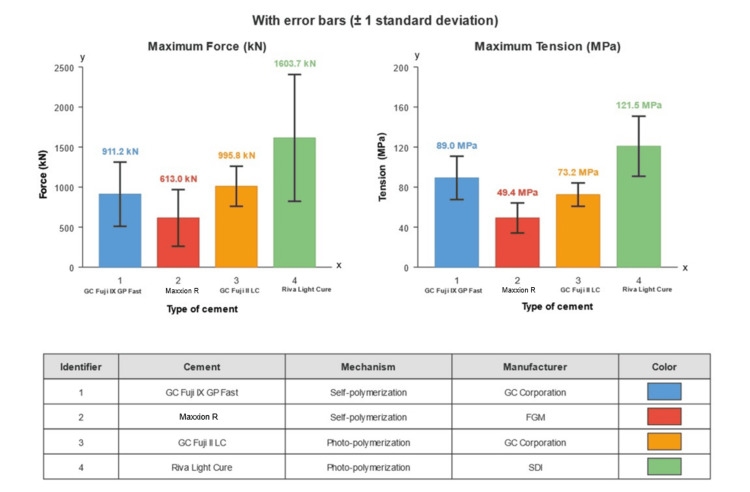
Comparison of force and maximum tension

Figure 1 shows a comparative analysis of maximum force and tension regarding the mechanical behavior of the four evaluated GICs. The results revealed a clear hierarchy in mechanical performance, where Riva Light Cure SDI stood out considerably above the other materials. This photopolymerizable cement exhibited values of maximum force (1,603.7 kN) and maximum tension (121.5 MPa).

Assumption testing and statistical approach

Before statistical analysis, data distribution was assessed for normality and homogeneity of variances. Shapiro-Wilk tests revealed significant deviations from normality for multiple groups: Maxxion R FGM (W = 0.892, p = 0.0108) and GC Fuji IX GP Fast (W = 0.901, p = 0.023), while GC Fuji II LC (W = 0.924, p = 0.078) and Riva Light Cure SDI (W = 0.919, p = 0.061) approached normal distribution. Levene's test indicated heterogeneity of variances (F = 4.23, p = 0.008), violating the assumption of homoscedasticity. These violations justified the use of the non-parametric Kruskal-Wallis test for group comparisons.

The analysis of dental materials using the Kruskal-Wallis test (Table 3) showed statistically significant differences between groups, with an H statistic of 58.408 and a p-value < 0.001 for both maximum force and maximum tension. This initial result indicated substantial differences in the mechanical behavior of the studied materials, which justified conducting pairwise comparisons to specifically identify between which groups these differences occurred.

**Table 3:** Kruskal-Wallis test **p < 0.001 (highly significant) ᵃKruskal-Wallis test ᵇGrouping variable: material type Large effect size (η² = 0.584) indicates substantial differences between groups

Test statistics	Maximum force (kN)	Maximum tension (MPa)
H of Kruskal-Wallis	58.408^a^	58.408^a^
Degrees of freedom (df)	3	3
Asymptotic significance (2-tailed)	<0.001**	<0.001**
Critical value (χ²_0.05,3_)	7.815	7.815
Effect size (η²)	0.584	0.584
Mean rank: GC Fuji IX GP Fast^b^	45.2	45.2
Mean rank: Maxxion R FGM^b^	19.1	19.1
Mean rank: GC Fuji II LC^b^	51.5	51.5
Mean rank: Riva Light Cure SDI^b^	80.2	80.2

Multiple pairwise comparisons (Table 4), performed with Bonferroni adjustment to control type I error, revealed specific patterns of differences between materials. Maxxion R FGM showed significant differences with all other materials: it presented significantly lower values compared to GC Fuji IX GP Fast GIC (p = 0.009), GC Fuji II LC (p < 0.001), and Riva Light Cure SDI (p < 0.001). These results positioned Maxxion R FGM as the material with the lowest resistance values in the study group.

**Table 4:** Pairwise comparisons between groups p < 0.01; **p < 0.001 Effect size r: small ≥ 0.1; medium ≥ 0.3; large ≥ 0.5 Each row tests the null hypothesis that the Sample 1 and Sample 2 distributions are equal Bonferroni correction applied for six comparisons (α = 0.05/6 = 0.0083)

Sample 1-Sample 2	Test statistic	Std. error	Std. test statistic	Sig.	Sig. adjusted	95% CI lower	95% CI upper	Effect size (r)
Maxxion R FGM-GC Fuji IX GP Fast	26	8.215	3.165	0.002**	0.009**	10.2	41.8	0.45
Maxxion R FGM-GC Fuji II LC	-32.283	8.049	-4.011	0.000**	0**	-47.8	-16.7	0.57
Maxxion R FGM-Riva Light Cure SDI	-61.083	8.049	-7.589	0.000**	0**	-76.6	-45.6	1.07
GC Fuji IX GP Fast-GC Fuji II LC	-6.283	8.049	-0.781	0.435	1	-21.8	9.2	0.11
GC Fuji IX GP Fast-Riva Light Cure SDI	-35.083	8.049	-4.359	0.000**	0**	-50.6	-19.6	0.62
GC Fuji II LC-Riva Light Cure SDI	-28.8	7.879	-3.655	0.000**	0.002**	-44.1	-13.5	0.52

At the upper end of performance, Riva Light Cure SDI proved to be significantly superior to all other materials. Significant differences were found compared to GC Fuji IX GP Fast GIC (p < 0.001), GC Fuji II LC (p = 0.002), and, as mentioned earlier, Maxxion R FGM (p < 0.001). This established Riva Light Cure SDI as the material with the highest resistance among all those evaluated.

The absence of significant differences between GC Fuji IX GP Fast GIC and GC Fuji II LC (p = 1.000) was also noted. These two materials showed statistically similar behavior, suggesting they could be interchangeable from the perspective of their resistance.

**Figure 2:**
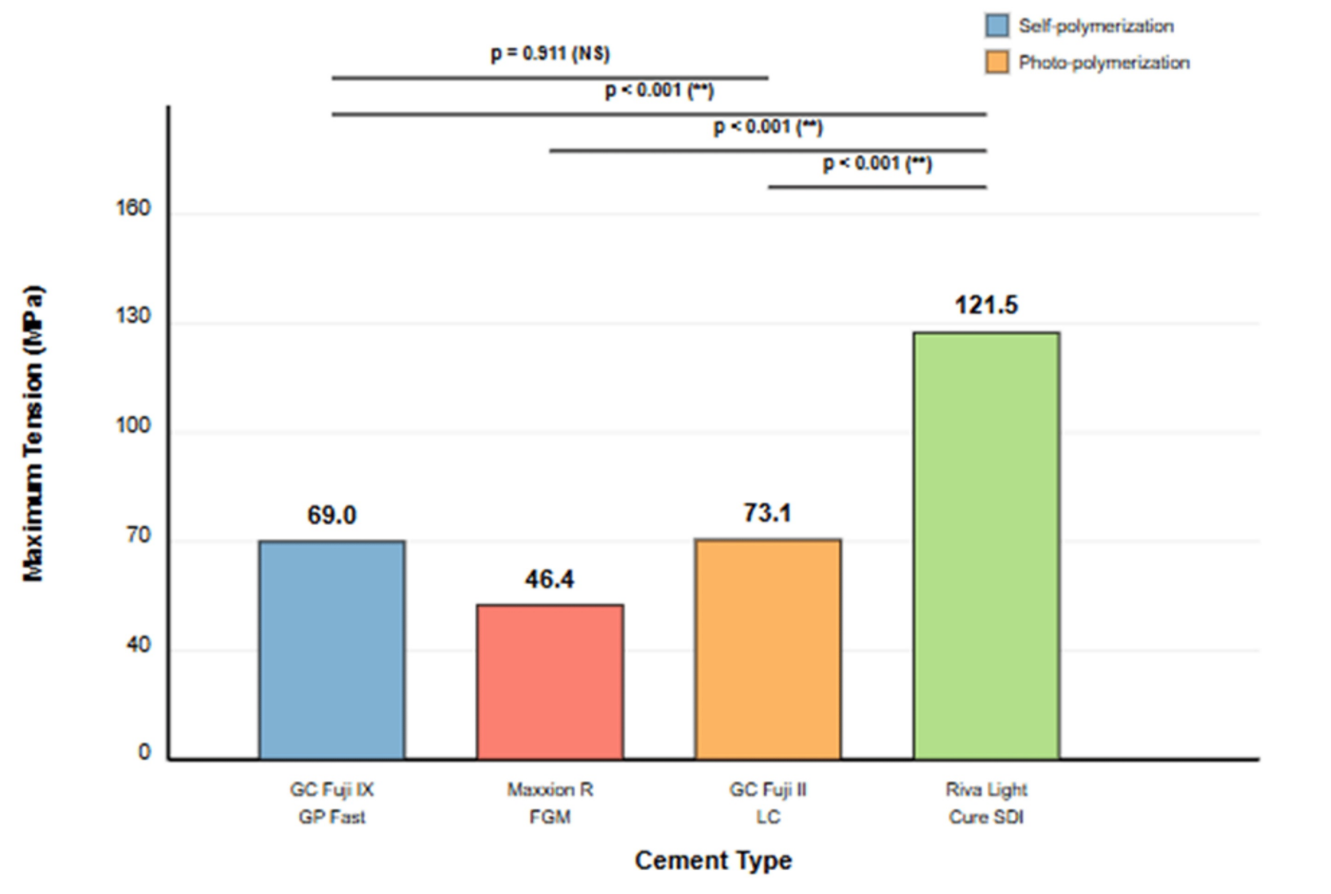
Comparison of maximum stress and its significant differences **Highly significant NS: not significant; ___: lines connecting compared groups

For better reference, Figure 2 shows the difference between the maximum tension values of the evaluated materials. Riva Light Cure SDI significantly stood out with a mean value of 121.5 MPa. This difference was not only statistically significant, as confirmed by statistical tests (Kruskal-Wallis) but also represented a clinically relevant advantage that could translate into greater longevity of restorations under tensional stress conditions.

At the intermediate level, GC Fuji II LC and GC Fuji IX GP Fast had values of 73.2 and 69.0 MPa, respectively. The proximity of these values, with a difference of only 6%, explained the absence of statistical significance between these two materials in post hoc tests.

At the lower end was Maxxion R FGM, with a value of 46.4 MPa, significantly below that of the other materials. This position placed it as the cement with the lowest tensile resistance, approximately 38% below the average of GC Corporation materials and 62% compared to Riva Light Cure SDI.

## Discussion

This study demonstrated significant differences in compressive and tensile strength among the four evaluated GICs. The statistically superior performance of Riva Light Cure SDI in both properties supports its suitability for restorations in high occlusal load areas, as previously reported [1]. However, its dependency on light-curing may pose clinical challenges in areas with limited access or with uncooperative pediatric patients.

The robust statistical approach employed in this study, including pre-specified effect size calculations (f = 0.4) and appropriate non-parametric testing due to normality violations, strengthens the validity of our findings. The enormous effect size observed (η² = 0.584) indicates that the differences between materials are not only statistically significant but also clinically meaningful, exceeding the pre-established threshold for clinical relevance.

Its superiority is likely due to the photopolymerization process, which creates a more homogeneous and densely cross-linked polymer matrix than the traditional acid-base reaction [1]. This curing method provides better control over setting time and results in improved mechanical properties [2]. However, the high variability observed (SD = 417.158 kN) may limit its clinical predictability, particularly when factors such as light exposure, curing distance, and time are not adequately controlled, emphasizing the need for strict curing protocols [3].

Interestingly, GC Fuji IX GP Fast GIC and GC Fuji II LC showed no significant difference in performance (p = 0.911), despite their distinct curing mechanisms. This finding contrasts with the assumption that light-cured materials are always superior [4] and instead supports the idea that composition and proprietary technologies may have a greater impact than the polymerization mode [5]. Clinically, this provides flexibility to use Fuji IX GP Fast in situations with limited access to curing lights or longer working times, without sacrificing strength.

Maxxion R FGM, while showing the lowest strength, presented the most consistent results, aligning with earlier reports that highlight certain self-cure ionomers for their predictability rather than peak strength [6]. This can be beneficial in clinical scenarios where consistency is prioritized [7].

The observed correlation between compressive and tensile strength supports previous findings, indicating shared structural mechanisms related to the inorganic-organic matrix [8]. This consistency across properties helps predict material behavior under various stress conditions.

The compressive strength of Riva Light Cure SDI (1,603.7 kN) notably exceeded earlier reports (around 1,200 kN), possibly due to advancements in chemical formulation and nanoparticle incorporation [9,10]. Clinically, this position modified glass ionomers, such as Riva, to make them viable alternatives for high-load areas and minimally invasive procedures.

The 15-day storage protocol in artificial saliva at 37°C differs from the 28-day standard used by other studies [11]. Still, it reflects a realistic post-restoration period during which the most significant mechanical changes occur [12]. The chosen method simulates early clinical performance, thereby supporting its real-world relevance.

Although statistical limitations, such as non-normality and heteroscedasticity, were detected (Shapiro-Wilk, Levene), the highly significant ANOVA result (p = 1.13E-19) validates the clinical relevance of the observed differences [11]. The deviation in normality for Maxxion R FGM (p = 0.0108) may be attributed to its unique composition or setting reaction, which affects the distribution of mechanical properties. This supports previous observations that specific ionomer components influence both the mean strength and variability, which are essential factors for clinical performance [13].

The maximum tensile strength results revealed a considerable gap between Riva Light Cure SDI (121.47 MPa) and the others. This may be related to differences in glass composition, especially the Al₂O₃/SiO₂ ratio and fluoride content [14]. Clinically, this supports the use of Riva in areas of high tension or extensive restorations. At the same time, lower-resistance materials, such as Maxxion R FGM, may be better suited to low-load applications that prioritize ease and predictability.

When interpreting these results, it is important to consider functional thresholds for restorations. Typical masticatory pressures rarely exceed 70 MPa in posterior areas [15], meaning both Riva Light Cure SDI and GC materials are mechanically sufficient for most clinical cases. In comparison, Maxxion R FGM (46.43 MPa) may be borderline in high-load situations.

These findings support the selection of tailored materials based on clinical needs. Materials can be categorized as high-performance (Riva Light Cure SDI), intermediate (GC Fuji IX GP Fast GIC and GC Fuji II LC), or basic (Maxxion R FGM), providing a practical framework for decision-making [16].

In pediatric dentistry, these distinctions are crucial. Challenges such as limited moisture control and short working times are common. The similarity in strength between GC Fuji IX GP Fast and GC Fuji II LC allows clinicians to choose the curing method based on the clinical context without compromising outcomes, providing a significant advantage in pediatric care [17].

Glass ionomers function as “smart materials,” further enhancing their clinical value [10]. Their ability to release and recharge fluoride offers additional therapeutic benefits, particularly in caries-prone patients. While this study focused on mechanical strength, future research may explore the correlation between strength and fluoride release.

Modern formulations are overcoming traditional mechanical limitations [18,19]. Riva Light Cure SDI's strength approaches that of some resin-based materials, narrowing the historical gap and expanding its clinical use. Although this study did not assess the effect of nanoparticle integration-an emerging area in ionomer development-it establishes a solid baseline for future investigations in that direction [20].

In summary, the evolving mechanical properties and bioactive potential of glass ionomers support their growing role in dentistry. These materials now offer a balance of strength, bioactivity, and ease of use, especially relevant in pediatric and minimally invasive practices [21].

This study was conducted under controlled in vitro conditions, which cannot fully replicate the complex dynamics of the oral environment, such as temperature fluctuations, masticatory forces, salivary composition, and pH variations. The 15-day storage period in artificial saliva, although clinically relevant for early performance assessment, does not account for long-term aging or wear. Only compressive and tensile strengths were evaluated, without assessing other clinically relevant properties such as fluoride release, wear resistance, or bond strength. Additionally, the results are limited to the specific commercial brands tested; extrapolation to other GICs should be approached with caution. Future research including longer observation periods, in vivo studies, and broader mechanical and bioactive property evaluations is warranted.

## Conclusions

The light-curing GICs showed greater compressive strength compared to the self-curing ones. Furthermore, this study reveals that specific chemical composition and manufacturer technology emerge as important variables when determining performance. The similarity between cements of the same brand, GC Fuji II LC (965.819 kN) and GC Fuji IX GP Fast (911.169 kN), was notable, with a difference of only 5.7% in their averages, despite using different polymerization mechanisms, compared to Riva Light Cure SDI (1,603.7 kN) being superior and Maxxion R FGM (612.984 kN) being the one with the lowest strength.

## References

[1] Chaudhary P, Sundaragiri KS, Shrimal D, Gehlot P (2023). Evolving trends in glass ionomer cement: from historical insights to nano-enhanced materials in restorative dentistry. Int J Sci Res.

[2] Correia da Silva DO, de Melo Silva I, de Oliveira Rocha A, dos Anjos LM, Oliveira Lima T, dos Anjos Santos RD (2021). Glass ionomer cement and its applicability in dentistry: a narrative review with emphasis on its properties. Res Soc Dev.

[3] Saridena US, Sanka GS, Alla RK, Ramaraju Av, Suresh SM, Raju Mantena S (2022). An overview of advances in glass ionomer cements. Int J Dent Mater.

[4] Sidhu SK, Nicholson JW (2016). A review of glass-ionomer cements for clinical dentistry. J Funct Biomater.

[5] Berg J (2002). Glass ionomer cements. Pediatr Dent.

[6] Junior LV, Barros AK, da Silva LH, Gaia LG, Binas IW, de Mendonca IC (2022). Glass ionomer cement: literature review. Braz J Health Rev.

[7] Messer-Hannemann P, Böttcher H, Henning S, Schwendicke F, Effenberger S (2023). Concept of a novel glass ionomer restorative material with improved mechanical properties. J Funct Biomater.

[8] Xie D, Zhao J, Weng Y, Park JG, Jiang H, Platt JA (2008). Bioactive glass-ionomer cement with potential therapeutic function to dentin capping mineralization. Eur J Oral Sci.

[9] Panetta A, Lopes P, Novaes TF, Rio R, Fernandes GV, Mello-Moura AC (2024). Evaluating glass ionomer cement longevity in the primary and permanent teeth-an umbrella review. J Funct Biomater.

[10] Zakaria AM, Kamaluddin NA, Hashim W, D'Agostino C (2024). Age-inclusive transit environments: an exploration of public transportation systems for elderly. Environ-Behav Proc J.

[11] Makanjuola J, Deb S (2023). Chemically activated glass-ionomer cements as bioactive materials in dentistry: a review. Prosthesis.

[12] Park EY, Kang S (2020). Current aspects and prospects of glass ionomer cements for clinical dentistry. Yeungnam Univ J Med.

[13] Croll TP, Nicholson JW (2002). Glass ionomer cements in pediatric dentistry: review of the literature. Pediatr Dent.

[14] Kłos J, Nicholson JW, Czarnecka B (2021). Glass-ionomer dental cements as novel solid-state buffers. J Mater Res Technol.

[15] Agha A, Parker S, Patel MP (2016). Development of experimental resin modified glass ionomer cements (RMGICs) with reduced water uptake and dimensional change. Dent Mater.

[16] Setiawan A, Gunawan JA, Wulansari S, Nugroho D (2021). Effect of nanofilled resin-based coating on the compressive strength of glass ionomer cement-in vitro. Maj Kedokt Gigi Indones.

[17] Chandru TP, Chandran S, Peedikayil FC, Kottayi S, Aparna TP, Aravind A (2023). Comparative evaluation of compressive strength of self-cure, dual-cure, and light-cure glass ionomer cements in a simulated oral environment: an in vitro study. Int J Clin Pediatr Dent.

[18] Creanor SL, Awawdeh LA, Saunders WP, Foye RH, Gilmour WH (1998). The effect of a resin-modified glass ionomer restorative material on artificially demineralised dentine caries in vitro. J Dent.

[19] Raghimi EC, Biglar N, Sadighian S, Karamitanha F, Nouri A, Nourian A (2024). Compressive strength and fluoride release profile of a glass ionomer cement reinforced with silver-hydroxyapatite-silica hybrid nanoparticles: an in vitro study. Int Orthod.

[20] Pérez-Castro B, Flores-Ledesma A, Rubio-Rosas E, Teutle-Coyotecatl B, Flores-Ferreyra BI, Argueta-Figueroa L, Moyaho-Bernal ML (2024). Comparison of the physical properties of glass ionomer modified with silver phosphate/hydroxyapatite or titanium dioxide nanoparticles: in vitro study. J Clin Pediatr Dent.

[21] Dionysopoulos D, Gerasimidou O, Papadopoulos C (2022). Modifications of glass ionomer cements using nanotechnology: recent advances. Recent Prog Mater.

[22] Thbayh KK, Albadr RM, Ziadan KM, Fiser B (2023). Expanding potential dental applications of glass ionomer cement by incorporating with nano-hydroxyapatite. Karbala Int J Mod Sci.

[23] Sidhu SK (2011). Glass-ionomer cement restorative materials: a sticky subject?. Aust Dent J.

[24] Bollu IP, Hari A, Thumu J (2016). Comparative evaluation of microleakage between nano-ionomer, giomer and resin modified glass ionomer cement in class V cavities-CLSM study. J Clin Diagn Res.

